# Association between gastric content fluidity and pars oesophageal ulcers in nursery pigs: a cross-sectional study of high-risk Danish herds using commercial feed

**DOI:** 10.1186/s40813-021-00199-x

**Published:** 2021-02-23

**Authors:** Juan Miguel Peralvo-Vidal, Nicolai Rosager Weber, Jens Peter Nielsen, Matthew Denwood, Svend Haugegaard, Anni Øyan Pedersen

**Affiliations:** 1 Department of Veterinary and Animal Sciences, Section for Production, Nutrition and Health, University of Copenhagen, Grønnegårdsvej 2, 1870 Frederiksberg C, Denmark; 2 SEGES Danish Pig Research Centre, Danish Agriculture and Food Council, Axeltorv 3, 1609 Copenhagen V, Denmark; 3 Department of Veterinary and Animal Sciences, Section for Animal Welfare and Disease Control, University of Copenhagen, Grønnegårdsvej 8, 1870 Frederiksberg C, Denmark; 4 Laboratory for Pig Diseases, SEGES Danish Pig Research Centre, Danish Agriculture and Food Council, Vinkelvej 13, 8620 Kjellerup, Denmark

**Keywords:** Gastric ulcer, Pars oesophagea, Nursery pig, Risk factor, Gastric content, Solid phase, Ad libitum feeding

## Abstract

**Background:**

The objective of this cross-sectional study was to assess the within-herd prevalence of pars oesophageal ulcers (POU) in high-risk Danish herds using commercial diets. Furthermore, we aimed to estimate the association between gastric content fluidity and POU using a generalised additive model (GAM). The study included 200 clinically healthy nursery pigs randomly selected from ten farms (20 pigs from each farm). The 10 farms were selected based on a suspected high prevalence of gastric ulcers. Post-mortem gastric ulcer assessment was based on macroscopic lesions, and gastric content fluidity was assessed based on the solid particle sedimentation percentage (solid phase).

**Results:**

We observed an overall prevalence of 35.5% for POU in nursery pigs. Within-herd prevalence varied considerably among farms, with values ranging from 0% in Farm 1 to 84% in Farm 4. Our model showed strong associations between POU and gastric content fluidity (*P* < 0.001), as well as between POU and farm of origin (*P* < 0.001). In addition, we observed that the risk of POU decreased non-linearly as the gastric content solid phase percentage increased, i.e. as the gastric content became more solid.

**Conclusion:**

We have demonstrated that pars oesophageal ulcers are present in Danish herds with nursery pigs fed commercial diets. Furthermore, we have established that gastric content fluidity is strongly associated with POU in nursery pigs. Even so, we cannot conclude that gastric content fluidity is solely responsible for POU. Future research should look into the association between pars oesophageal ulcers and both farm management activities and individual pig factors.

**Supplementary Information:**

The online version contains supplementary material available at 10.1186/s40813-021-00199-x.

## Background

Porcine gastric ulceration is a prevalent disease primarily reported in finisher pigs and sows around the world [1, 2]. The disease etiology is multifactorial, but is associated with an increased fluidity of the gastric content [3, 4]. Risk factors such as pelleted feed, ad libitum feeding, small feed particle size, and starvation are known to predispose pigs to gastric ulceration in finisher pigs [5–7].

Reports on this disease date back as early as 1950, yet identifying pigs with gastric ulcers remains challenging due to the absence of clinical signs [6, 8]. This is particularly true in the case of nursery pigs, where the diagnosis of gastric ulceration is generally only feasible post mortem at the abattoir. As a result, prevalence and risk factor assessment for gastric ulceration is only available for finisher pigs and sows. Although, both nursery pigs and finisher pigs are fed and raised similarly, little is known about the prevalence of gastric ulcers or the effect of gastric content fluidity on gastric ulceration in nursery pigs.

Porcine gastric ulcers are principally located in the non-glandular area of the stomach, in the pars oesophagea at the cardiac region [9]. Pigs with pars oesophageal ulcers (POU) predominantly present a highly fluid gastric content with a disrupted or non-existent pH layering [4, 10]. This high fluidity of the gastric content allows direct contact of the pars oesophagea with a low pH gastric content and high concentrations of pepsin, which is normally restricted to the fundus of the stomach [9, 11]. Since the stratified squamous epithelium of the pars oesophagea does not produce mucus, hyperplasia then develops as a result of prolonged exposure to the low pH and pepsin enzymatic activity resulting from highly fluid gastric content [7, 12].

Gastric ulcers in pigs have been reported in all continents, with the prevalence in finisher pigs ranging from 32 to 65% [1]. In contrast, gastric ulcers in nursery pigs are barely described in the available scientific literature. This may be because ulcers cannot be diagnosed antemortem under production conditions, or because nursery pigs are not frequently observed in abattoirs. To the best of our knowledge, the occurrence of POU in nursery pigs under commercial production conditions and the association between gastric content fluidity and POU in this age group have not previously been studied.

The primary objective of this study was to estimate the within-herd prevalence of POU in nursery pigs in ten high-risk Danish farms using commercial diets fed ad libitum. The secondary objective was to estimate the association between gastric content fluidity, measured as the solid particle sedimentation percentage, and pars oesophageal ulceration in nursery pigs.

## Materials and methods

### Study design and study population

This cross-sectional study was carried out in ten commercial Danish farms selected by convenience in December 2017. The inclusion criteria for farms included historic records of gastric ulceration in finisher pigs or sows, the use of commercially produced feed, and ad-libitum feeding. From each of the ten farms, 20 clinically healthy nursery pigs were selected by systematic random sampling from 15 to 20 different pens. There was no sample size consideration for this study; a sample size of 20 nursery pigs was chosen because it was the number of pigs that the researcher and one animal technician were able to handle at each farm visit. Random selection was intentionally carried out in sections with the oldest nursery pigs. The 200 selected nursery pigs were all DanBred (Landrace × Yorkshire × Duroc). To reduce sampling bias, the same researcher carried out the selection process.

### Data collection and gastric ulcer assessment

Pigs were euthanised by bleeding through sectioning of the jugular vein and carotid artery after stunning by captive-bolt pistol in accordance with Danish regulations for euthanasia of animals [13]. On each farm, nursery pigs were sampled within the same day from 8:00 AM to 12:00 AM. There was no feed withdrawal for all sampled pigs. All nursery pigs remained in the nursery pen with ad libitum feed access until sampling. Body weight and sex were recorded post mortem. To avoid the gastric content from spilling, stomachs were removed from the abdominal cavity, preserving 5 cm of duodenum and oesophagus. Upon arrival at the Laboratory for Pig Diseases, an experienced pathologist assessed the stomach health according to macroscopic lesions, based on Nielsen and Ingvartsen’s scoring system [14]. According to these lesion scores, stomachs with no lesions, parakeratosis, or erosions were classified as “No pars oesophageal ulcers” (NPOU) and stomachs with ulcers, oesophageal stenosis, and scars were classified as “Pars oesophageal ulcers” (POU). Gastric content fluidity was assessed based on the solid particle sedimentation percentage (solid phase). This involved measuring the gastric content fluidity using graduated plastic beakers after 24 h at 4 °C. Only stomachs with gastric content were included in this assessment.

### Feeding and feed particle analysis

Samples of approximately 4 kg of feed were collected at each farm from a minimum of six different feeders or from the silos during feed loading. Then 100 g (± 25 g) was obtained from each sample using a sample riffle splitter (© 2019, Pfeuffer GmbH). Feed particle size was assessed according to Mikkelsen and collaborators (2004) for wet sieve analysis of pelleted feed [15]. Sieves (Retsch®) measuring 3150 μm, 2000 μm, 1400 μm, 1000 μm, 500 μm, and 355 μm, and a wet sieve shaker set to an amplitude of 1.5 mm (Retsch® AS 200) were used for this purpose. Feed particle size was expressed as average particle size in mm (AVP), geometric mean diameter in μm (GMD), and geometric standard deviation (GSD). GMD and GSD were calculated based on the equation by Wilcox and collaborators (1970) [16]. In all visited farms commercial feed was formulated according to the Danish nutrition standards for nursery pigs and was based on wheat, barley and soybean meal [17].

### Statistical analysis

The relationship between gastric ulceration (dichotomous outcome) and gastric content fluidity (solid phase percentage) was estimated using a generalised additive model (GAM) with binary response and logistic link function. Gastric content fluidity was fit using a smoothing term (thin plate regression spline), sex was fit as a fixed effect (females relative to males), and herd was fit as a random effect as descrived in Additional files 1 and 2. Entire males and castrated pigs were included as one single group. Statistical analysis was performed using R version 3.6.0 [18]. The mgcv package version 1.8–31 [19] was used to fit the GAM, and the tidyverse package [20] was used to extract output and produce plots. Odds Ratios were calculated based on the GAM fit using the function *or_gam* from the package oddsratio version 2.0.1 [21]. Posterior 95% confidence intervals for prevalence estimates were calculated using a Bayesian method based on a conjugate Beta(1,1) prior, with Highest Posterior Density Intervals calculated using the TeachingDemos package version 2.12 [22].

## Results

Descriptions of the farms and feed particle size assessments are shown in Table 1. Farm size is presented as the annualized inventory of sows per year and the number of 30 kg pigs produced per year. The study included both small (180 sow years) and large herds (1100 sow years). Feed particle size expressed as both GMD and in mm did not vary considerably across farms. Summary statistics for gastric ulceration assessment in 200 nursery pigs are presented in Table 2, alongside the macroscopic lesion score per farm. Summary statistics for the independent variables sex (females, entire males, and castrates), body weight at sampling (kg), and sedimentation of solid particles (%) are presented in Table 3.

**Table 1:** Summary of farm characteristics, production capacity, and assessment of feed particle size

Farm	Herd characteristics					Feed type	Particle size assessment				
	No. of sows per year	No. of 30 kg pigs produced per year	x̅ No. of nursery pigs / pen	x̅ m2 / nursery pig	x̅ Temperature nursery room in °C		GMD (μm)	GSD (μm)	< 1 mm (%)	1–2 mm (%)	> 2 mm (%)
1	180	6000	33	0.35	19	Expanded meal feed	627.8	2.27	66.7	19.7	12.6
2	535	16,000	29	0.37	18	Pelleted	540.7	1.92	76.4	20.8	2.7
3	850	28,000	22	0.49	19	Pelleted	635.7	2.25	67.6	19.6	12.8
4	240	7400	35	0.33	22	Crushed pellets (Liquid form)	486	1.83	82.2	15.3	2.5
5	1100	35,000	32	0.31	18	Pelleted	620.8	2.32	69.1	16	14.9
6	400	14,000	19	0.35	18	Pelleted	532.9	2.09	76.7	15.2	8
7	370	12,000	24	0.35	20	Pelleted	584.7	2.12	71.2	18.8	10
8	400	11,000	19	0.46	17	Pelleted	565.8	2.08	72.8	19.9	7.3
9	630	20,000	32	0.37	18	Pelleted	483.3	1.75	83.3	16	0.7
10	700	23,000	29	0.38	18	Crushed pellets (Liquid form)	464.6	1.73	84.6	15	0.4

*GMD* Geometric mean diameter, *GSD* Geometric standard deviation

**Table 2:** Within-herd prevalence and summary statistics for gastric lesion assessment in 200 nursery pigs^a^ from 10 farms

Farm	No. of observations	No Pars Oesophageal Ulcers (NPOU)			Pars Oesophageal Ulcers (POU)			Within-Herd Prevalence (No.)	95% CI
		Healthy	Parakeratosis	Erosion	*Ulcer*	*Scar*	*Oesophageal stenosis*		
1	20	18	2	0	0	0	0	0%	0.87; 1.00
2	20	4	9	2	1	4	0	25% (5)	0.54; 0.89
3	20	6	8	1	3	2	0	25% (5)	0.54; 0.89
4	20	0	3	0	7	8	2	85% (17)	0.04; 0.34
5	20	3	4	1	7	2	3	60% (12)	0.21; 0.60
6	20	5	9	1	2	2	1	25% (5)	0.54; 0.89
7	20	4	5	0	6	4	1	55% (11)	0.25; 0.65
8	20	11	8	0	1	0	0	5% (1)	0.79; 0.99
9	20	5	8	0	3	4	0	35% (7)	0.46; 0.82
10	20	0	11	1	4	4	0	40% (8)	0.39; 0.78
Total	200	56	67	6	34	30	7	35.5%^b^ (71)	–

^a^ Danish system for gastric health assessment [14] ^b^ Overall prevalence in the study

**Table 3:** Descriptive statistics for independent observations in 200 nursery pigs from 10 farms

Independent variable	Unit	Count	Percent	SD
Sex	Females	87	44%	–
	Entire males	12	6%	–
	Castrates	101	51%	–
Pig weight at sampling	Kg	22.41	–	4.71
Solid phase % – POU nursery pigs	Average	61.70	–	13.93
Solid phase % – NPOU nursery pigs	Average	85.29	–	16.71

### Prevalence of pars oesophageal ulcers

The overall and within-herd prevalence is presented in Table 2. Pars oesophageal ulcers were found in a total of 35.5% (range: 5 to 84%) of nursery pigs (71 nursery pigs in total). There was a considerable variation in the prevalence of POU at herd level (Table 2), for example Farm 1 had no pigs with POU, while Farm 8 had one (5%) and Farm 4 had 17 (85%). In this study, POU were identified in all farms where nursery pigs were given commercial pelleted feed (Farm 2 to 10; Tables 1 and 2), while nursery pigs at Farm 1 were given an expanded meal feed and did not present POU.

### Association between gastric content fluidity and gastric ulceration

**Fig. 1:**
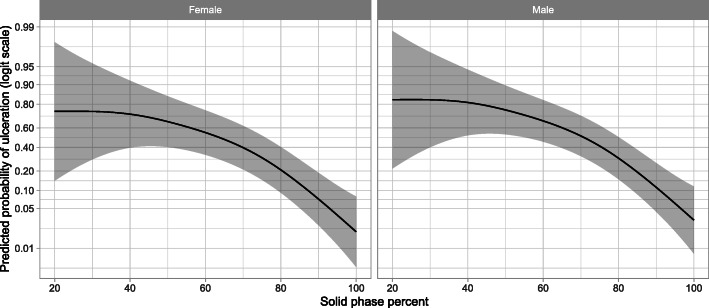
Estimated relationship between gastric content fluidity and the probability of gastric ulceration in nursery pigs (Female and Male), estimated using a generalised additive model (using a smoothing term). The grey shaded area represents 95% confidence intervals for the relationship

In this study, 13 stomachs were empty at sampling and were excluded from the statistical analysis. Our generalised additive model showed that there was a strong association between gastric content fluidity (*P* < 0.001) and POU (Tables 3 and 4). This model also demonstrated a decrease in the odds of having POU as the gastric content solid phase percentage increased following a non-linear pattern (Fig. 1). Indeed, the protective effect of solid gastric content (with low fluidity) is only apparent when the gastric content solid phase percentage increases above the range 36.8 to 52.6% (OR 0.69, 95% CI: 0.48; 0.99; Table 5 and Fig. 1). However, our model identified a strong association between gastric ulceration in nursery pigs and both gastric content fluidity and the farm of origin (*P* < 0.001; Table 4). In addition, we did not observe an association between sex and POU in our model (Table 4 and Fig. 1).

**Table 4:** Generalised additive model with binary response and logistic link function for evaluating the risk of POU in 200 nursery pigs, with farm as a random effect

Parameter	Type	Coefficients	EDF	*P*-value
Intercept	Fixed	−1.63	–	< 0.001
Sex (Males)	Fixed	0.5	–	NS
Farm	Random	–	5.44	< 0.001
^a^Solid phase	Thin plate regression spline	–	2.74	< 0.001

^a^ Solid particle sedimentation percentage from the gastric content after 24 h at 4 °C. NS Non-Significant. EDF Effective Degrees of Freedom

**Table 5:** Odds Ratio for POU according to the solid phase percentage as predicted based on the generalised additive model (20% quantiles)

Parameter	Solid phase range (%)		Odds Ratio	95% CI
Solid phase	21%	36.8%	1.56	0.49; 4.97
	36.8%	52.6%	0.69	0.48; 0.99
	52.6%	68.4%	0.46	0.43; 0.49
	68.4%	84.2%	0.21	0.20; 0.21
	84.2%	100%	0.09	0.12; 0.07

### Assessment of feed particle size

Table 1 shows the feed particle size analysis expressed both as GMD and the percentage distribution of particle size in mm. Particle size varied to a lesser degree among the farms: Farm 4, Farm 9, and Farm 10 had the smallest particle sizes (< 486 μm GMD), while Farm 3 had the largest particle size in this study at 635.7 μm GMD. Interestingly, Farm 1, with a 0% prevalence of POU and using expanded meal feed, had a feed particle size of 627.8 μm GMD, which is no larger than that found for Farm 3 with 25% POU prevalence and pelleted feed.

## Discussion

In this study, we observed an overall prevalence of 35.5% for pars oesophageal ulcers in clinically healthy nursery pigs. In addition, we identified a significant association (*P* < 0.001) between pars oesophageal ulceration and both gastric content fluidity (solid phase percentage) and farm of origin (*P* < 0.001). To the best of our knowledge, this is the first time that the prevalence of gastric ulceration and the role of gastric content fluidity have been assessed in nursery pigs under commercial conditions.

The prevalence presented here is comparable to results observed in finisher pigs at abattoir level in Australia (30%), Colombia (34.8%), USA (32%), and Denmark (30%) [1, 7, 23, 24]. However, we cannot assume that this prevalence applies to nursery pigs in general because we only focused on high-risk herds with commercial diets fed ad libitum and with previous cases of gastric ulceration in finisher pigs or sows. Therefore, the prevalence presented here only reflects the within-herd prevalence on these study farms.

We showed that increased gastric content fluidity (solid phase percentage) is associated with an increased risk of pars oesophageal ulcers in nursery pigs. This result is in accordance with previous observations by various researchers, who found that the physical characteristics of the feed (i.e. finely ground) and an increase fluidity of gastric content were associated with gastric ulceration [4, 10, 25]. Our model also showed an association between gastric ulcers and the farm of origin. However, this observation might be the result of only including 20 nursery pigs per farm.

The within-herd prevalence ranged from 0% for Farm 1 to severely affected herds with a prevalence of 85 and 60% for Farm 4 and Farm 5, respectively. Risk factor studies on finisher pigs have shown that feed type (i.e. pelleted feed) can have a negative effect on pars oesophageal health [7, 26]. The variation in within-herd prevalence that we observed might be associated with the feed type, since Farm 1 used expanded meal feed, while Farm 4 and Farm 5 used pelleted feed. This is in accordance with the protective effect of meal feed on pars oesophageal ulceration previously reported in finisher pigs [27]. Interestingly, the feed used in Farm 1 was also expanded, which is associated with an increased risk of gastric lesions in finisher pigs [28]. Therefore, we cannot conclude that the sole reason for Farm 1 having no observed pars oesophageal ulcers was due to feed type. Although the genetic pool in Danish farms is highly homogeneous (DanBred), genetic factors might also be associated with the variation in gastric ulceration among farms [29, 30]. Furthermore, it is also possible that within-herd variation for gastric ulceration is the result of subclinical disease status in some nursery pigs that might have prompted some level of anorexia days before sampling. It has previously been reported that a reduced feed consumption and feed withdrawal are associated with a higher risk for gastric ulceration in finisher pigs [31, 32]. Therefore, these observations suggest that additional factors at farm or individual pig level might also play an important role in POU development.

## Conclusion

The risk of pars oesophageal ulceration was significantly higher in nursery pigs with a highly fluid gastric content than in nursery pigs with a solid gastric content (reduced fluidity). However, we are not able to conclude that gastric content fluidity alone is responsible for pars oesophageal ulceration. Pars oesophageal gastric ulceration in nursery pigs appears to be a recurrent problem in herds using commercial pelleted feed. The 35.5% overall prevalence exclusively reflects the level of gastric ulceration in the selected study herds, yet the extent of this problem varied considerably among herds. We observed nursery pigs with healthy stomachs or with only minor lesions in herds with a high prevalence of gastric ulcers; despite them all sharing the same environment, feed, and management practices. This implies that individual pig and farm management activities might also play an important role in the development of gastric ulceration.

## Supplementary Information

**Additional file 1.** Statistical GAM model (r file).

**Additional file 2.** Raw data for the statistical GAM model.

## Data Availability

Gastric ulceration assessment data sets and R scripts for this study are available in the following files:

## Abbreviations

POU: Pars Oesophageal Ulcers
GAM: Generalised additive model
AVP: Average particle size in mm
GMD: Geometric mean diameter in μm
GSD: Geometric standard deviation in μm
